# Development and validation of parenting measures for body image and eating patterns in childhood

**DOI:** 10.1186/s40337-015-0043-5

**Published:** 2015-03-14

**Authors:** Stephanie R Damiano, Laura M Hart, Susan J Paxton

**Affiliations:** School of Psychology and Public Health, La Trobe University, Melbourne, 3086 Australia

**Keywords:** Parent, Children, Body image, Eating patterns, Validity, Knowledge

## Abstract

**Background:**

Evidence-based parenting interventions are important in assisting parents to help their children develop healthy body image and eating patterns. To adequately assess the impact of parenting interventions, valid parent measures are required. The aim of this study was to develop and assess the validity and reliability of two new parent measures, the Parenting Intentions for Body image and Eating patterns in Childhood (Parenting Intentions BEC) and the Knowledge Test for Body image and Eating patterns in Childhood (Knowledge Test BEC).

**Methods:**

Participants were 27 professionals working in research or clinical treatment of body dissatisfaction or eating disorders, and 75 parents of children aged 2–6 years, who completed the measures via an online questionnaire. Seven scenarios were developed for the Parenting Intentions BEC to describe common experiences about the body and food that parents might need to respond to in front of their child. Parents ranked four behavioural intentions, derived from the current literature on parenting risk factors for body dissatisfaction and unhealthy eating patterns in children. Two subscales were created, one representing positive behavioural intentions, the other negative behavioural intentions. After piloting a larger pool of items, 13 statements were used to construct the Knowledge Test BEC. These were designed to be factual statements about the influence of parent language, media, family meals, healthy eating, and self-esteem on child eating and body image. The validity of both measures was tested by comparing parent and professional scores, and reliability was assessed by comparing parent scores over two testing occasions.

**Results:**

Compared with parents, professionals reported significantly higher scores on the Positive Intentions subscale and significantly lower on the Negative Intentions subscale of the Parenting Intentions BEC; confirming the discriminant validity of six out of the seven scenarios. Test-retest reliability was also confirmed as parent scores on the two Parenting Intentions subscales did not differ over time. Eleven out of the 13 Knowledge Test items demonstrated sufficient discriminant validity and test-retest reliability.

**Conclusions:**

Overall, results indicated that the six-scenario Parenting Intentions BEC and the 11-item Knowledge Test BEC are valid and reliable measures for parents of young children.

**Electronic supplementary material:**

The online version of this article (doi:10.1186/s40337-015-0043-5) contains supplementary material, which is available to authorized users.

## Background

A growing literature supports the important role that parents can play in the development of children’s body size attitudes (e.g., [1]) and eating patterns (e.g., [2-4]), as they are formed in early childhood. Children as young as four years old already report a strong weight bias, attributing negative characteristics to large figures and positive characteristics to thin figures [1]. In addition, eating patterns, such as emotional overeating, have been shown to be stable from ages four to 11 [5]. Given that weight bias and unhealthy eating patterns may lead to body image and eating problems later in life (e.g., [6,7]), it is important that parents are aware of ways in which they can help protect their child against the development of these problems.

It has been suggested that parents may increase the risk of, or protect against, the development of body image and eating concerns in their children in a number of direct and indirect ways (e.g., [8,9]). Direct parental behaviours can include commenting to a child about their weight or appearance, including teasing or pressuring them to lose weight, or encouraging a child to diet. Direct parental comments about a child’s weight and shape have been associated with higher levels of drive for thinness and body dissatisfaction in children (e.g., [8,10]). Parental teasing about a child’s weight has also been found to have an effect on girls’ and boys’ body dissatisfaction [11], and one study found that parental teasing had a moderate association with drive for muscularity in boys [12]. In addition, a longitudinal study of children followed from birth to 11 years found that parental pressure for the child to be thin and parental over-control of their child’s eating were linked to a child’s drive for thinness, having a thin body preoccupation, and perceived social pressure to be thin [13]. Parental encouragement to diet has also been associated with high levels of body dissatisfaction in children [10]. Indirect parental behaviours typically involve a parent’s actions or attitudes that are not necessarily intended to impact the child. Specifically, negative parental comments about their own bodies may model self-criticism and encourage children to judge themselves and others based on appearance [9,14]. In addition, parental engagement in excessive exercise or dieting may emphasise the importance of adhering to cultural body size ideals [15,16].

It is important to acknowledge that although parents play an important role in shaping children’s attitudes and behaviours, they are not the only source of influence. Research also demonstrates that media, which often conveys unrealistic and unachievable body size ideals, can influence a child’s body size attitudes, body image, and eating concerns [17,18]. Specifically, in a study of 5- to 8-year-old girls, Dohnt and Tiggemann [19] found that exposure to appearance-focused television predicted a decrease in appearance satisfaction. Moreover, exposure to television soaps and music videos has been related to higher internalisation of the thin ideal, body dissatisfaction, and restricted eating in 7- to 9-year-old girls [20]. Although media is not specifically a parental factor, parents can have some control over what media their young children may be exposed to; thus, parents can play an important role in influencing their child’s media exposure.

In light of the evidence that parents play an important role in increasing or mitigating childhood risks of body dissatisfaction and eating problems, it is important to develop prevention programs for parents of preschool-aged children [21]; however, valid measures are necessary to assess the impact of such prevention efforts. A recent systematic review by Hart, Cornell, Damiano, and Paxton [21], concluded that data for only a very small number of interventions for parents of school-age children exist, and only two have been found to be effective in modifying parent behaviours relevant to child body image and eating problems. Notably, the effects of including parents in body dissatisfaction and eating disorder prevention programs have largely not been analysed, and there is a need for useful and sensitive measures of parents’ influences on children’s body dissatisfaction and disordered eating risk [21]. Validated measures of parental feeding practices, such as the Child Feeding Questionnaire [22] and Pre-schooler Feeding Questionnaire [23] have been developed and are widely used. Such measures assess how parents *feed* their child, and the influence of parental feeding practices on children’s eating behaviours and weight have been analysed [4,24]. There are, however, no validated measures that assess parent knowledge and behaviours relevant to both body image *and* healthy eating patterns in young children.

The aim of this study was to develop and assess the psychometric properties of two new parent measures developed for the purpose of assessing parent behaviours and knowledge about children’s body image and eating patterns: the Parenting Intentions for Body image and Eating patterns in Childhood (Parenting Intentions BEC) and the Knowledge Test for Body image and Eating patterns in Childhood (Knowledge Test BEC). To do this, their discriminant validity [25] was assessed by comparing responses from parents in the community with responses from body image and eating disorder professionals, to determine whether the proposed measures could discriminate between expert and community-level behavioural intentions and knowledge; a common method used in the development of eating disorder assessments [26]. It was hypothesised that professionals would report more positive and less negative parenting behavioural intentions and higher Knowledge Test scores than the community-based parents. Second, test-retest reliability assessed whether the measures could produce stable responses from a community sample of parents over time. It was hypothesised that parenting behavioural intentions and knowledge would not change across time as parents would not be subject to an intervention between tests. If found to be valid and reliable, these measures may be used in future intervention evaluations, which are greatly needed to help parents of pre-schoolers prevent the development of body dissatisfaction and unhealthy eating patterns in childhood.

## Methods

### Study procedure and participants

Following ethics approval from the La Trobe University Human Research Ethics Committee, parent and professional participants were recruited using the methods and inclusion criteria detailed below. Informed consent and responses to questionnaires were securely completed online using Qualtrics survey software.

#### Parents

Participants were a community sample of 75 parents (71% mothers), recruited from Early Childhood Services (e.g., childcare centres, preschools, play groups, and maternal and child health centres) and social network sites (e.g., Facebook) throughout Victoria, Australia. Parents were required to be over the age of 18 years, have at least one child between the ages of two and six years, and live in Victoria. Parents were aged between 27 and 47 years (*M* = 37.68, *SD* = 4.01), were generally well-educated with 85.4% reporting having completed a university degree, and most frequently identified as having an Australian ethnic background (49.3%). The majority of parents reported currently working or studying (50.7%), while 32.0% reported both working and home duties, and 17.3% reported home duties only. Parents reported having between one and four children, with the majority having two children (59.2%). Parents completed a baseline questionnaire and then completed the retest questionnaire approximately eight weeks later (*M* = 8.26, *SD* = 2.56).

#### Professionals

Professionals were 27 experts working in the fields of body image, eating disorders, parenting, or obesity as researchers, teachers, or clinicians. Professionals were recruited from Australia, Canada, Ireland, New Zealand, the United Kingdom, and the United States, via an emailed invitation to participate. Potential professional participants were identified through their association with professional organisations, such as the Academy for Eating Disorders, or through their authoring of relevant scientific or clinical resources identified in a systematic literature review [21]. Professionals were also identified through their association with parenting organisations, training organisations, or authoring of relevant parenting or prevention materials. Professionals were required to have a minimum of five years clinical, research, or training experience in at least one of the relevant fields. Of the 27 professionals who participated, 96.3% were females, and were aged between 31 and 70 years (*M* = 46.15, *SD* = 11.17). Regarding their expertise, 22.2% were research only, 25.9% were clinical work only, 11.2% were education/training only, and 40.7% reported belonging to multiple categories. The majority of professionals (74.1%) reported their area of expertise as being across multiple categories, while 18.5% reported their expertise as eating disorders, 3.7% as parenting, and 3.7% as body image. For years working in their area of expertise, 44.4% reported between five and 10 years, and 55.6% reported working 11 or more years.

### Measures

Participants completed a brief demographic questionnaire before completing the two new measures. Demographic questions for parents included their gender, age, highest level of education, occupational status, ethnicity, and number of children. Demographic questions for professionals included their gender, age, type of expertise (e.g., research, clinical work, education/training), area of work (e.g., eating disorders, parenting, obesity, prevention, body image), and their work affiliation (e.g., clinical service, university).

#### Parenting intentions BEC

The Parenting Intentions BEC was designed to measure parents’ behavioural intentions relevant to body image and eating issues in young children (see Additional file 1). Its development and validation was modelled on the Mental Health Literacy Survey [27]. The Mental Health Literacy Survey assesses behavioural intentions towards people with mental illness by presenting a vignette describing an individual with symptoms of a mental disorder, and asking participants about their intentions for action. Vignettes were developed in line with DSM-IV and ICD-10 psychiatric criteria and the responses of mental health professionals and lay members of the public were compared to determine discriminant validity.

For the Parenting Intentions BEC, seven domains of parenting behaviours identified by experts as being likely modifiable risk factors for the development of child body dissatisfaction and disordered eating were chosen for measurement. These seven domains were overlapping outcomes from two previous studies; a review of the academic literature on parenting strategies that represent proposed modifiable risk factors for the development of body dissatisfaction or eating disorders, and a Delphi expert consensus study [28]. The review was performed as part of a systematic literature search of studies reporting intervention and evaluation projects [21]. The Delphi expert consensus study developed guidelines on parenting to prevent body dissatisfaction and unhealthy eating patterns in preschool children [28]. For the purposes of developing the new measure of parenting intentions, seven domains were selected for measurement from the overlap between the risk factor review and the Delphi study. The seven domains were: (1) teaching your child to value difference in appearance; (2) helping your child to accept their appearance and respect their body; (3) discouraging appearance-based teasing or bullying; (4) counteracting negative media messages; (5) encouraging your child to value personal qualities not related to appearance; (6) teaching your child healthy eating patterns; and (7) teaching your child about a healthy body rather than weight. For each domain, a scenario describing a parent–child interaction was developed, and these were informed by real-life situations reported by parents of young children in a separate focus group study [Hart et al.: What parents know and want to learn about healthy eating and body image in preschool children: Findings from focus group and structured interviews, submitted].

For each scenario, four parenting responses were developed. Participants were asked to rank these potential responses from 1 (*most likely to respond to the scenario in this way*) to 4 (*least likely to respond to the scenario in this way*). Again, these different parenting responses were derived from the literature review of parenting risk factors and the results of the Delphi study [28]. The four response options included: (1) a positive parenting response, taken from the current research evidence suggesting the behaviour was likely to be protective against a child developing body dissatisfaction and disordered eating; (2) a response that would indicate a lack of knowledge, designed to represent how a parent with an inadequate understanding of child body image and healthy eating might behave; (3) a negative parenting response, taken from the current evidence suggesting the behaviour was likely to increase the risk of a child developing body dissatisfaction and disordered eating by increasing stigmatising attitudes towards weight, shape, or appearance; and (4) an unhelpful parenting response, designed to represent an unhelpful strategy for dealing with the situation, but one that was unlikely to increase weight, shape, or appearance stigma. To ensure an appropriate level of comprehension, items were subject to the Flesch-Kincaid Grade Level test. It received a score of 6.0 that suggested that items are likely to be understood by an average 6th grade student.

#### Scoring

From the ranking of these parenting strategies, two subscales were calculated, the Positive Intentions subscale and the Negative Intentions subscale. The Positive Intentions subscale was derived by scoring one point for each occasion that parents ranked the positive response first and one point for each occasion parents ranked the negative response last. A sum of scores was calculated, such that the total score ranged between 0 and 14. Higher scores represented more positive behavioural intentions, which are thought likely to promote *healthy* body image and eating patterns in young children. The Negative Intentions subscale was calculated by scoring two points for each occasion parents ranked the negative response first or one point when ranked in second position. A sum of scores was calculated, whereby the total score ranged between 0 and 14. Higher scores represented more negative behavioural intentions that are thought likely to promote *unhealthy* body image and eating patterns in young children.

#### Knowledge Test BEC

The Knowledge Test BEC was designed to measure parents’ knowledge relevant to body image and eating issues in young children (see Additional file 1). The Knowledge Test BEC was developed using similar methods to those adopted in the development of the Mental Health Knowledge Schedule [29]. Evans-Lacko et al. [29] conducted an extensive literature review to ensure that the knowledge being assessed in each item was supported by the research evidence as potentially influencing mental health attitudes and behaviours. An item pool was generated and subsequently pilot tested [29]. During the piloting of the scale, Evans-Lacko et al. [29] also sought feedback on the wording and comprehensibility of items. Additionally, Cohen and Swerdlik [30] emphasise the importance of developing an item pool at least double the number of items of the final scale and then subsequently pilot testing the items with a sample who resemble the population for which the scale is designed for; the method also adopted in the present study.

The development of the Knowledge Test BEC was based on the review of literature of parenting strategies that have been identified as likely modifiable risk factors for the development of body dissatisfaction or eating disorders, performed as part of a systematic literature search of studies reporting intervention and evaluation projects [21]. Initially, a pool of 39 items was developed by the authors from the risk factor literature, to reflect parental attitudes and behaviours that may be helpful in promoting healthy body image and eating patterns in children. The items were designed to be factual statements about the influence of parent language, media, family meals, healthy eating, and self-esteem on child eating patterns and body image, which could be rated by participants on a 4-point rating scale ranging from 1 (*false*) to 4 (*true*). A 4-point rating scale was chosen over the traditional 2-point (true or false) rating scale, because it gives participants the opportunity to provide nuanced ratings that expresses the *strength* of their knowledge, yet still forces a direction to their response as no midpoint (e.g., unsure) option is offered [31].

These items were pilot tested with a convenience sample of 16 parents (68.8% mothers; *M*_*age*_ = 36.63, *SD* = 4.99) of young children aged between one and 12 years, from Melbourne, Australia. Parents reported having between one and five children (*M* = 2.06, *SD* = 1.06). The purpose of the questionnaire pilot was to identify the ease of responding correctly to items and to receive feedback on the items. In addition to the 4-point rating scale, there was an option for parents to indicate whether an item was difficult to respond to due to the unclear meaning of an item or where they thought an item’s wording was vague, and were asked to explain why it was difficult or vague. Individual item analysis revealed that for 10 (25.6%) items 100% of parents scored correctly. Therefore, these items were removed from the scale. In addition, 16 (41.0%) of the items were reported as difficult or vague and were therefore removed. The content of the remaining 13 items was then compared with key strategies for parents of pre-schoolers to prevent body dissatisfaction and unhealthy eating patterns [32], which were determined using the Delphi expert method [28], to ensure the remaining items closely matched the content of expert guidelines. To ensure an appropriate level of comprehension, items were subjected to the Flesch-Kincaid Grade Level test. It received a score of 6.7 that suggested that items were likely to be understood by an average 6th to 7th grade student.

#### Scoring

To provide a total score of knowledge, two scoring methods were trialled. First, the 4-point rating scale was scored by reverse scoring the six items whereby false was the correct response (see Additional file 1). Then all items were summed to provide a total score between 13 and 52, whereby higher scores indicated a greater level of knowledge relevant to body image and healthy eating patterns in children. Second, the traditional 2-point (true or false) rating scale was imposed on the data to test the difference in variability of responses. Along with the *true* and *false* response options, the *somewhat* categories were collapsed into either correct or incorrect responses, such that if a statement was true, participants who responded with either *somewhat true* or *true*, scored one point. Seven items were positively worded in this way. If a statement was false, participants who responded either *false* or *somewhat false* scored one point. Six items were negatively worded in this way. Each correct response was allocated one point, and scores were then summed to provide a total score between 0 and 13, whereby higher scores indicated a greater level of knowledge.

### Data analysis

Tests of normality for the two Parenting Intentions subscales and the Knowledge Test 2-point rating scale showed significant skew that was not amenable to transformation; thus, non-parametric analyses were subsequently conducted. The Knowledge Test 4-point rating scale was normally distributed, thus, parametric tests were conducted. The discriminant validity for the Parenting Intentions and Knowledge Test BEC was established by selecting only the items that were able to discriminate between the expert knowledge of the professionals and the lay knowledge of the parents. This method has been used in previous research assessing the psychometric properties of eating disorder literacy measures [33]. As a first step, each item on the Knowledge Test BEC and Positive Intentions subscale of the Parenting Intentions BEC were required to meet the following criteria to be subject to further analyses: (a) correctly answered by a large majority of professionals (>70%); and (b) the proportion of professionals correctly answering the item was greater than the proportion of parents who answered the item correctly [30]. In addition, to assess the Negative Intentions subscale of the Parenting Intentions BEC, a minority of professionals (<30%) were required to rate the negative response as their first (most likely) response.

Discriminant validity was then established by conducting Mann–Whitney *U* tests (non-parametric) to determine the statistically significant difference in parents’ and professionals’ mean scores on the Positive Intentions subscale, Negative Intentions subscale, and Knowledge Test 2-point rating scale. An equivalent parametric test, independent samples *t*-test, was conducted to determine group differences on the Knowledge Test 4-point rating scale, as this scale was normally distributed. Subsequently, using parent data only, the intra-class correlation coefficient (ICC) was calculated to assess the test-retest reliability of the two Parenting Intentions subscales and two scoring methods of the Knowledge Test BEC. Wilcoxon Signed Rank Tests (non-parametric) were then conducted to ensure that scores on the three non-normally distributed scales did not change over time. An equivalent parametric test, paired samples *t*-test, was used to examine the change over time on the Knowledge Test 4-point rating scale.

## Results

### Discriminant validity

The frequencies of professionals’ and parents’ responses to the Parenting Intentions BEC and Knowledge Test BEC items are provided in Tables 1 and 2. It was evident that Scenario 4 from the Parenting Intentions BEC did not perform well, as few professionals correctly rated the positive response first, and a greater proportion of parents rated the positive response first. Scenario 4 was therefore removed from the measure and subsequent analyses. On the basis of this change, scores on the Positive and Negative Intentions subscales now ranged between 0 and 12. For the Knowledge Test, a greater proportion of parents than professionals scored correctly on Items 8 and 9; thus, these two items were removed from the measure and subsequent analyses. Subsequently, total scores on the Knowledge Test 4-point rating scale ranged between 11 and 44, and on the 2-point rating scale ranged between zero and 11.

**Table 1:** **Proportion of participants who ranked the positive and negative response first for the Parenting Intentions BEC**

**Item**	**Proportion positive response first** ^**a**^		**Proportion negative response first** ^**b**^	
	**Professionals**	**Parents**	**Professionals**	**Parents**
Scenario 1	100%	61.3%	0%	9.3%
Scenario 2	100%	90.7%	0%	2.7%
Scenario 3	100%	89.3%	0%	5.3%
Scenario 4	48.10%	62.7%	29.6%	30.7%
Scenario 5	81.50%	69.3%	7.4%	10.7%
Scenario 6	74.10%	62.7%	0%	2.7%
Scenario 7	100%	89.3%	0%	4.0%

^a^the proportion of professionals and parents who rated the positive strategy as their most likely response in each scenario.

^b^the proportion of professionals and parents who rated the negative strategy as their most likely response in each scenario.

**Table 2:** **Proportion of participants who responded correctly to the Knowledge Test BEC**

**Item**	**Professionals**	**Parents**
Item 1	85.2%	65.3%
Item 2	88.9%	68.0%
Item 3	96.3%	94.7%
Item 4	96.3%	81.3%
Item 5	100%	85.3%
Item 6	96.3%	92.0%
Item 7	100%	96.0%
Item 8	92.6%	93.3%
Item 9	96.3%	98.7%
Item 10	96.3%	93.3%
Item 11	100%	85.3%
Item 12	96.3%	76.0%
Item 13	96.3%	77.3%

A Mann–Whitney *U* Test for the Positive Intentions subscale revealed that professionals (*Md* = 9, *n* = 27) reported significantly more positive behavioural intentions than parents (*Md* = 8, *n* = 75), *U* = 577.00, *z* = −3.35, *p* = .001, *r* = .33. A Mann–Whitney *U* Test for the Negative Intentions subscale revealed that professionals (*Md* = 0, *n* = 27) reported significantly less stigmatising behaviours than parents (*Md* = 1, *n* = 75), *U* = 732.50, *z* = −2.22, *p* = .027, *r* = .22. A Mann–Whitney *U* Test for the Knowledge Test 2-point rating scale revealed that professionals (*Md* = 11, *n* = 27) reported significantly greater knowledge of parenting strategies to promote healthy body image and eating patterns than parents (*Md* = 10, *n* = 75), *U* = 514.50, *z* = −3.93, *p* < .001, *r* = .39. An independent samples t-test for the Knowledge Test 4-point rating scale revealed that professionals (*M* = 41.00, *SD* = 1.90) reported significantly greater knowledge than parents (*M* = 36.31, *SD* = 4.79), *t* (99) = −7.07, *p* < .001. The magnitude of the differences in the means (mean difference = −4.69, 95% *CI*: −6.01 to −3.38) was large (η^2^ = .33).

### Test-retest reliability

The ICC was calculated to assess the test-retest reliability of responses for 68 (90.7%) of the community sample of parents, who completed the measures on two occasions. Kruskal-Wallis tests showed that there were no differences in scores at baseline, between parents who completed both the baseline and retest questionnaires, and those who only completed the baseline questionnaire. The ICC showed moderate agreement between baseline (T1) and retest (T2) for the Positive Intentions subscale (*M*_*T1*_ = 7.68, *SD*_*T1*_ = 2.13; *M*_*T2*_ = 7.79, *SD*_*T2*_ = 1.82; *ICC* = .59, *p* < .001), good agreement for the Negative Intentions subscale (*M*_*T1*_ = 1.57, *SD*_*T1*_ = 1.76; *M*_*T2*_ = 1.59, *SD*_*T2*_ = 1.48; *ICC* = .69, *p* < .001), and excellent agreement for the Knowledge Test 2-point rating scale (*M*_*T1*_ = 9.18, *SD*_*T1*_ = 1.85; *M*_*T2*_ = 9.32, *SD*_*T2*_ = 1.61; *ICC* = .90, *p* < .001) and 4-point rating scale (*M*_*T1*_ = 36.38, *SD*_*T1*_ = 4.72; *M*_*T2*_ = 36.31, *SD*_*T2*_ = 4.42; *ICC* = .88, *p* < .001), according to the criteria outlined in McDowell [34]. Wilcoxon Signed Rank Tests revealed no significant changes across time for the Positive Intentions subscale (*z* = −.07, *p* = .94), the Negative Intentions subscale (*z* = −.35, *p* = .73), or the Knowledge Test 2-point rating scale (*z* = −1.20, *p* = .23). A paired samples *t*-test for the Knowledge Test 4-point rating scale revealed no significant changes from test to retest, *t* (67) = 0.20, *p* = .84.

## Discussion

The aim of this research was to develop and validate two new measures of parents’ behaviours and knowledge relevant to the development of children’s body image and eating problems. The six-scenario Parenting Intentions BEC, encompassing the Positive Intentions and Negative Intentions subscales, and the 11-item Knowledge Test BEC were found to be valid and reliable measures, supporting their use in the evaluation of much needed prevention interventions for parents of pre-schoolers.

The Parenting Intentions BEC is an innovative assessment approach that uses brief vignettes of parent–child situations involving the body and food to assess parents’ behavioural intentions that may protect against, or increase the risk of, the development of children’s body and eating problems. Initial validity checks of individual items to ensure that professionals demonstrated more positive behavioural intentions than parents, indicated that six scenarios from the Parenting Intentions BEC appropriately discriminated expertise from lay knowledge. Discriminant validity was established for the two Parenting Intentions subscales by comparing the scores of the professionals and parents. Although parents generally demonstrated positive strategies towards promoting positive body image and healthy eating patterns in their young children, the subscale scores clearly demonstrated that professionals reported more positive and less stigmatising parenting intentions. Notably, there was also room for improvement for the parent’s positive behavioural intentions with the aim to protect children against the development of body image and eating problems. The test-retest reliability of the Parenting Intentions BEC subscales was also established. Significant correlations between parents’ baseline and retest scores were found, and there was a lack of any significant changes on either subscale, indicating that the Parenting Intentions subscales provide stable results over multiple testing occasions.

Scenario 4 was removed from the Parenting Intentions BEC following the initial validity checks. Scenario 4 described a situation in which a pre-schooler wanted to watch Top 40 music videos. When constructing this item, the authors considered these age-inappropriate media to be viewed by pre-schoolers, even when co-watched with a parent, due to research findings that suggest music videos often contain highly sexualised content [35,36]. Hence, the most positive response was considered to be for a parent to change the channel to a program with age-appropriate viewing; however, less than 50% of professionals rated the positive parenting strategy as their most likely response. This scenario was therefore removed from the final scale and subsequent analyses. In a recent Delphi consensus study with experts in the field of body image, eating disorders, and parenting, there was a lack of expert consensus over the potential negative influence of music videos, specifically, on young children [28]. This lack of expert consensus may help to explain why so few professionals in the present study ranked the positive strategy first.

The Knowledge Test BEC is a novel measure developed to assess parent knowledge of proposed risk and protective factors pertaining to preschool-aged children’s body image and eating patterns. Two scoring methods were examined for the final 11-item Knowledge Test BEC to determine whether a 4-point true-false rating scale would better capture knowledge than the traditional 2-point true/false method. Discriminant validity was established for both scoring methods, with professionals demonstrating greater knowledge of parenting strategies than parents with large effect sizes. The reliability of the Knowledge Test BEC was also established using both methods of scoring as significant correlations between parents’ baseline and retest scores were found, and there was a lack of any significant changes over time. Importantly, however, the 2-point rating scale showed a ceiling effect with parents overall reporting a median Knowledge Test score of 10 out of 11, while the 4-point rating scale demonstrated a normal distribution of scores, more variability, and no ceiling effect in responses. Given the stronger statistical properties of the 4-point scale and the need for a measure of parenting knowledge about children’s body image and eating patterns that allows room for improvement following an intervention, it is suggested that future use of the Knowledge Test BEC include use of the 4-point rating scale.

The initial validity checks of individual items from the Knowledge Test, to ensure that professionals demonstrated a higher level of knowledge than parents, indicated that 11 items from the Knowledge Test BEC appropriately discriminated expertise from lay knowledge. Items 8 and 9 were removed from the Knowledge Test due to a higher proportion of parents responding correctly to the items than professionals. Interestingly, a high proportion of parents correctly rated Item 8 as false (‘Giving a child food for good behaviour is a healthy way to encourage them to behave’). This is in line with previous research that has identified that mothers of young children use instrumental feeding to promote eating of healthy foods or finishing a meal, rather than to encourage good behaviour per se [37]. It was not surprising that a high proportion of parents rated Item 9 correctly (‘Early childhood is not too early for parents to help build a child’s body confidence’), as it is likely that there was some participant selection bias as a result of volunteers for this research being community-based parents with an interest in body image in young children. This sample, therefore, may be more inclined to perceive early childhood as an appropriate time to start promoting body confidence.

The small sample size of professionals is a limitation of this research. Previous research, comparing professional and community samples, has however established validity and reliability with smaller samples (e.g., [33]), and this study was able to demonstrate statistically significant differences between the professional and parent groups, suggesting that sample size did not impact on the power to detect medium to large effects. In addition, because the professional sample was recruited across a range of countries and disciplines, they can be considered to represent a range of expertise and knowledge, despite being small in number. Another possible limitation of the study was that the proportion of professionals who are parents is unknown; however, given that the aim was to assess their professional knowledge of body image and eating patterns in young children, this was not deemed to influence the results.

Given that parents were generally well-educated, the generalizability of results to the broader national or international parent community is somewhat limited. Thus, these new measures require validation in other cultures and languages before use, as the current sample was English speaking and well-educated, which was appropriate for the validation purposes of this study. It is possible that if parents scored lower on measures (e.g., less educated parents), the discriminant validity of the measures would increase when compared with professionals; thus, increase the room for improvement in parent responses, which would not interfere with interpretation of the present findings. Given that the parenting measures were designed to evaluate the impact of prevention programs for parents to reduce the risks of their children developing body dissatisfaction and eating problems, the ability of the new measures to discriminate scores of professionals and well-educated parents only further supports the strength of the measures.

## Conclusions

Previous research has highlighted that parents play an important role in guiding their child’s development of body image and eating patterns. There is a need to implement evidence-based prevention interventions to assist parents to provide a positive body image and eating environment for their children, and there is an accompanying need to develop valid measures of parenting variables that may be the target for intervention. The present findings demonstrate that the six-scenario Parenting Intentions BEC and 11-item Knowledge Test BEC are valid and reliable measures. Given that the measures discriminate between expert and community samples, and remain stable over time, our findings suggest that these instruments will adequately meet the requirements of intervention evaluation research, which requires room for intervention participant scores to improve over time.

## Additional file

Additional file 1:
**Appendix A.** The Parenting Intentions for Body image and Eating patterns in Childhood. **Appendix B.** The Knowledge Test for Body image and Eating patterns in Childhood.
