# Supplementary material for: Development and validation of parenting measures for body image and eating patterns in childhood
Source: J Eat Disord. 2015 Mar 14;3:5. doi: 10.1186/s40337-015-0043-5 (PMC4359773; doi:10.1186/s40337-015-0043-5)
Supplement: Additional file 1: — Appendix A. The Parenting Intentions for Body image and Eating patterns in Childhood. Appendix B. The Knowledge Test for Body image and Eating patterns in Childhood. [file 40337_2015_43_MOESM1_ESM.pdf]

Additional file 1

Appendix A

The Parenting Intentions for Body image and Eating patterns in Childhood

For each of the following scenarios please rank the items according to how likely you would be to react in the way described, with your young child. Please rank from MOST likely (1) to LEAST likely (4). Note that we do not expect you to have experienced each scenario, but ask that you think about what you might do if it occurred.

Scenario 1

In the supermarket, your child points to an overweight person and asks “why are they so big?”	Rank<br>1 2 3 4	*Item Identifier*
You explain to your child that everyone is different and that is OK.		*Positive*
You ignore the comment because you’re not really sure how best to tackle it.		*Lack of Knowledge*
You comment on the size of the person and explain to your child that it’s not healthy.		*Negative*
You think this is a good opportunity to tell your child about the importance of keeping trim and watching what they eat to avoid getting big.		*Unhelpful*

Scenario 2

You see your child look at themself in the mirror and say they hate their nose.	Rank<br>1 2 3 4	*Item Identifier*
You ask your child why they have said that and listen to what they have to say.		*Positive*
You don’t know what to say, so say nothing.		*Lack of Knowledge*
You tell your child that it doesn’t matter because they can change it when they are older.		*Negative*
You don’t worry about it because they are too young to have serious concerns about their appearance.		*Unhelpful*

Scenario 3

Your child is bigger than average. They come home from playing at their cousin's house and say that they were called "fat".	Rank<br>1 2 3 4	*Item Identifier*
You reassure your child that body size doesn't matter, as long as they are healthy.		*Positive*
You tell your child to just forget about it because you're not sure how to respond.		*Lack of Knowledge*
You suggest to your child that given the cousin's big size they shouldn't be making such comments.		*Negative*
You start limiting how much your child is eating to help them lose weight.		*Unhelpful*

Scenario 4 – removed from final scale

Your child wants to watch the latest Top 40 music videos on TV.	Rank<br>1 2 3 4	*Item Identifier*
You change the channel because you want to limit your child's exposure to media that isn't age-appropriate.		*Positive*
You think it should be suitable because it's PG rated.		*Lack of Knowledge*
You let them watch it and encourage them to dance along with you.		*Negative*
You allow your child to watch the videos, watch it with them, and comment on how the celebrities have unrealistic bodies.		*Unhelpful*

Scenario 5

An old friend of yours drops in, bringing along their child who is the same age as yours. You notice they don't play well together. After they leave, you ask your child why. Your child says because they thought the other child's freckles made them look funny.	Rank<br>1 2 3 4	*Item Identifier*
You explain to your child that they should try to play together again because you know they will find the visiting child is very kind.		*Positive*
You aren't quite sure what to say, so tell your child to be nicer next time.		*Lack of Knowledge*
You agree with your child that freckles do look funny, but they should still try to play nicely with other children.		*Negative*
You tell your child off for being mean.		*Unhelpful*

Scenario 6

You are at a party where there are lots of lollies, cakes, and sweets but no fresh food available. You would like to make sure your child doesn't eat too many of these foods.	Rank<br>1 2 3 4	*Item Identifier*
You allow your child to choose a small number of foods to enjoy, explaining that we need to leave room for dinner later on.		*Positive*
You follow what other parents are letting their children do.		*Lack of Knowledge*
You explain to your child that the party foods are bad for them and they might gain weight if they eat too many of them.		*Negative*
You allow your child to eat what they want now, but make sure they don't have any party foods for a while to make up for what they eat now.		*Unhelpful*

Scenario 7

You are concerned that your child is overweight.	Rank<br>1 2 3 4	*Item Identifier*
You try to give more nutritious foods and encourage the whole family to have time each evening where they are physically active together, without telling your child you are concerned about their weight.		*Positive*
You aren't sure what to do, so wait to see what happens.		*Lack of Knowledge*
You discuss how eating too many of the wrong foods will cause them to become overweight and they should try to eat less.		*Negative*
You encourage them not to eat as many snacks and make sure they don’t help themselves to second servings at mealtimes.		*Unhelpful*

Note: The Item Identifier column is for scoring purposes only; please remove when asking parents to complete the measure. It is further suggested that response options be presented in random order. A copy of the measure and scoring protocol is available from the authors.

Appendix B

The Knowledge Test for Body image and Eating patterns in Childhood

Please read each of the following statements and rate according to **how true** you think each statement is.	**False**	**Somewhat False**	**Somewhat True**	**True**
**1.** Teaching a child about how media manipulates images of celebrities is unlikely to help a child develop their own body confidence.**	1	2	3	4
**2.** A child's 'body image' refers to the way a child looks, or their physical appearance.**	1	2	3	4
**3.** Criticising the way another person dresses can encourage a child to worry about their appearance.	1	2	3	4
**4.** If your child was overweight, you should encourage them to feel good about the body they have.	1	2	3	4
**5.** Dieting is a healthy eating behaviour.**	1	2	3	4
**6.** Saying "I feel fat" in front of a child could be harmful to their development.	1	2	3	4
**7.** Regular family meals can protect a child from developing disordered eating.	1	2	3	4
**8.** Giving a child food for good behaviour is a healthy way to encourage them to behave.** **removed from final scale**	1	2	3	4
**9.** Early childhood is not too early for parents to help build a child's body confidence. **removed from final scale**	1	2	3	4
**10.** If a child is upset, parents should avoid giving them a special food to help them feel better.	1	2	3	4
**11.** Parents need not be concerned about how a child feels about their body until early adolescence.**	1	2	3	4
**12.** Parents should judge when their child is full.**	1	2	3	4
**13.** Characters in children's cartoon and movies can teach children that being thin or muscular will lead to success and happiness.	1	2	3	4

Note: **Reverse scored items. Underlining indicates the correct responses; please remove when asking parents to complete the measure. A copy of the measure and scoring protocol is available from the authors.
